# Synthesis and anti-rheumatoid arthritis activities of 3-(4-aminophenyl)-coumarin derivatives

**DOI:** 10.1080/14756366.2021.1873978

**Published:** 2021-02-08

**Authors:** Yuhang Miao, Jie Yang, Yinling Yun, Jie Sun, Xiaojing Wang

**Affiliations:** Institute of Materia Medica, Shandong First Medical University & Shandong Academy of Medical Sciences, Jinan, China

**Keywords:** 3-(4-Aminophenyl)-coumarin, rheumatoid arthritis, RA-FLSs proliferation inhibition, anti-inflammation

## Abstract

Rheumatoid arthritis is a chronic systemic disease characterised by an unknown aetiology of inflammatory synovitis. A large number of studies have shown that synoviocytes show tumour-like dysplasia in the pathological process of RA, and the changes in the expression of related cytokines are closely related to the pathogenesis of RA. In this thesis, a series of novel 3-(4-aminophenyl) coumarins containing different substituents were synthesised to find new coumarin anti-inflammatory drugs for the treatment of rheumatoid arthritis. The results of preliminary activity screening showed that compound **5e** had the strongest inhibitory activity on the proliferation of fibroid synovial cells, and it also had inhibitory effect on RA-related cytokines IL-1, IL-6, and TNF-α. The preliminary mechanism study showed that compound **5e** could inhibit the activation of NF-κB and MAPKs signal pathway. The anti-inflammatory activity of compound **5e** *in vivo* was further determined in the rat joint inflammation model.

## Introduction

Rheumatoid arthritis (RA) is the most common chronic inflammatory joint disease and a progressive autoimmune disease^1^. The main pathological feature of RA is the recurrent chronic inflammation in the synovial tissue of the joint, which gradually infiltrates the cartilage and subchondral bone in the joint, and significantly affects the joints, organs, and systems, and eventually leads to irreversible joint deformities and dysfunction^2^. At present, the research on the pathogenesis of RA is still a top priority^3^. During the pathological process of RA, synovial cells exhibit tumour-like abnormal proliferation, and invade into cartilage and bone, producing a variety of cytokines, leading to chronic inflammation of cartilage and bone^4^. There are two types of synovial cells: macrophage-like type A cells, with filamentous pseudopodia, invagination of serosa, vesicles, mitochondria, lysosomes, cytoplasmic fibres, and Golgi bodies, which have phagocytic function^5^; type B fibroblast-like synoviocytes (FLSs), which have a high concentration of endoplasmic reticulum structure. RA-FLS is an important effector cell in the course of RA. The over proliferation of RA-FLSs will lead to damage of joint and chronic persistent inflammation^6–9^. Therefore, inhibiting the proliferation of RA-FLSs is an important therapeutic strategy in the clinical treatment of RA. There are four main types of drugs for clinical treatment of RA: non-steroidal anti-inflammatory drugs such as aspirin^10^, glucocorticoid drugs such as Prednisone^11^, disease-altering drugs such as methotrexate (MTX)^12^, and biological agents such as TNF-α inhibitors^13^^,^^14^. Most of these RA treatment drugs have many side effects, and the biological agents are relatively expensive, leading to a huge burden on patients and delay of disease treatment. Therefore, it is imperative to develop new anti-RA drugs with high efficiency, low cost, and few side effects.

**Scheme 1.**
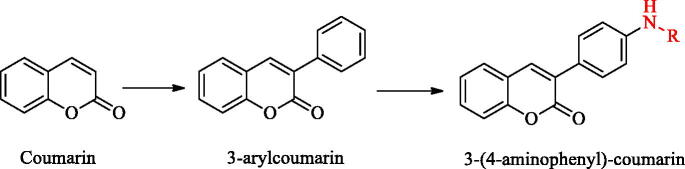
Structural derivation of 3-(4-aminophenyl)-coumarins.

Coumarin (called Benzopyran one or o-hydroxycinnamic acid-8-lactone), which is composed of fused benzene and α-pyranone ring, is a kind of rich phenolic derivatives^15^^,^^16^. Coumarins have a variety of biological activities including anti-tumour^17^, antibacterial^18^, antioxidant^19^, anti-Alzheimer’s disease^20^, and anti-inflammation^21^. There have been many reports on the anti-inflammatory activity of coumarin derivatives, and the relationship between structure and activity of (SAR) has also been explored, it was found that the introduction of the electron-rich hydrophobic group into the 3-position of coumarin nucleus could enhance the anti-inflammatory activity^22^^,^^23^. The therapeutic effect of coumarin on RA has been reported before^24^. In recent years, many coumarin derivatives obtained from natural products related to the treatment of RA have been found, such as Daphning, a coumarin derivative extracted from natural product Daphne odora Var. It has a good therapeutic effect on autoimmune arthritis^25^. Synthetic coumarin derivatives are also widely used as in anti-RA. It has been found that scavenging reactive oxygen species, inhibiting the activation of NF-jB and the synthesis of TNF-α can effectively treat RA^26^^,^^27^; Multi-target drugs (such as NSAIDs, CORMs) and carbonic anhydrase inhibitors (CAI) can be used as hybrid compounds to treat RA through synergy, which is an important research direction for anti-RA drugs in the future^28^^,^^29^. Carbonic anhydrase (CAs) isoforms are overexpressed in the synovium of RA patients and there is the interaction between various CAs subtypes and arthritis-like diseases, so carbonic anhydrase inhibitors have been paid more and more attention in the development of anti-RA drugs^30^. Coumarin derivative is a kind of suicided CAIs, which is hydrolysed by esterase CA activity and binds to the active site of carbonic anhydrase, so coumarin has a unique and new carbonic anhydrase inhibition mechanism^31^^,^^32^. 3-(4-aminophenyl)-coumarin derivatives are derived from 3-arylcoumarin, and the positional isomer structure of 3-arylcoumarin is similar to that of flavonoids and isoflavones. From the structure-activity relationship analysis, the electron-rich hydrophobic group introduced at position 3 can enhance the anti-inflammatory activity, and the hydroxyl group at position 7 or 8 can interact with the receptor through an intermolecular hydrogen bond. Joint inflammation can lead to elevated ROS, which can erode bones and joints and cause pain. The persistent pain behaviour in RA is related to the increase of inflammatory mediators in ROS. Flavonoids have the effect of anti-oxidation and relieving pain^33^. Therefore, we speculate that 3-(4-aminophenyl)-coumarin derivatives may also have antioxidant imbalance activity. We tend to explore the anti-RA activity of 3-(4-aminophenyl)-coumarin derivatives through structural extension, which provides a new type of coumarin structure for the treatment of RA, but also a new direction for structural modification of coumarin compounds (Scheme 1).

## Results and discussion

### Synthesis of novel 3-(4-aminophenyl)-coumarin compounds containing various substituents

**Scheme 2.**
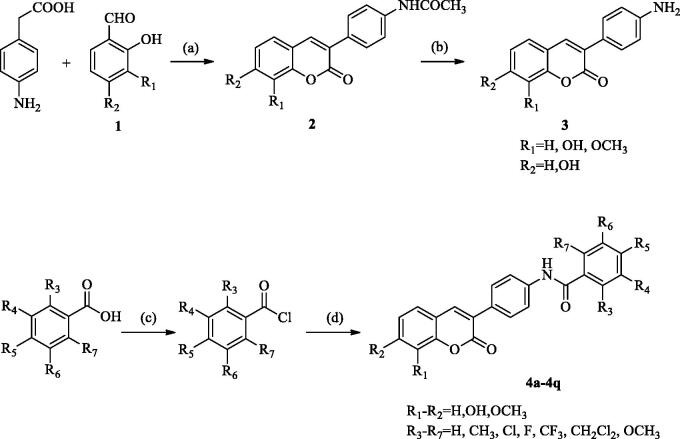
The synthetic route of 3-(4-aminophenyl)-coumarins 3 and target product 4. Reagents and conditions: (a) acetic anhydride, Et_3_N, 115 °C. (b) HCl ethanol. (c) COCl_2_, DCM, reflux. (d) 3-(4-aminophenyl)-coumarin 3, acetone, pyridine, and RT.

**Scheme 3.**
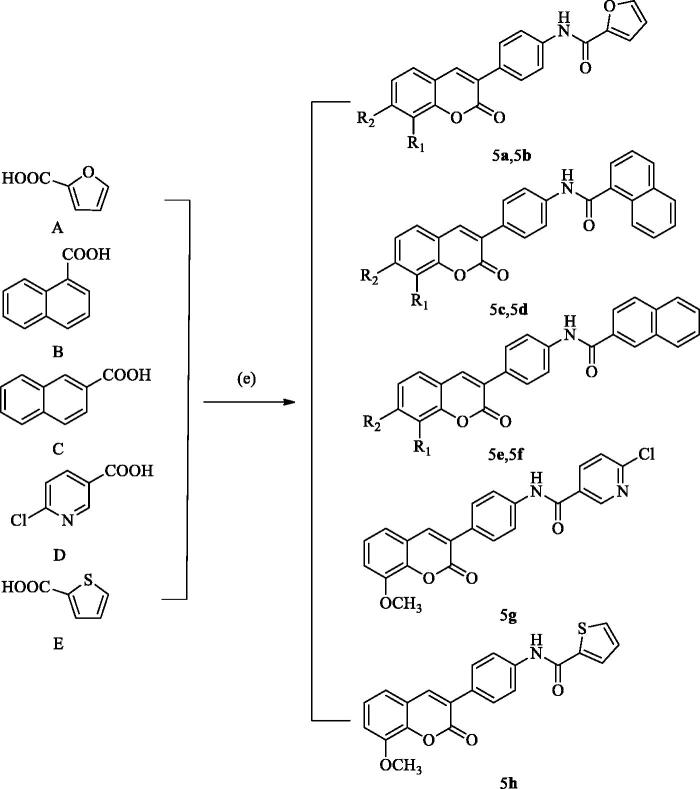
General synthetic route to compounds **5a–5h**. Reagents and conditions: (e) 3-(4-aminophenyl)-coumarin 3, DCC, HOBt, toluene, and RT.

The 3-(4-aminophenyl)-coumarin derivatives were synthesised using the synthesis method previously reported by our group, and the synthesis route is shown in Scheme 2. The intermediate 2 was synthesised from substituted p-amino-phenylacetic acid and o-hydroxybenzaldehyde by Perkin reaction and acidified with hydrochloric acid to give 3-(4-aminophenyl)-coumarin intermediate 3. In the next step, a series of substituted benzoyl chlorides were synthesised from benzoic acid. The target compound was obtained by amide condensation of 3 with substituted benzoyl chloride in the mixed solution of pyridine and acetone (Table 1, **4a–4q**). The synthetic route of 3-(4-aminophenyl)-coumarin derivatives with heterocycles is shown in Scheme 3. Hydroxy benzotriazole (HOBt) and dicyclohexylcarbodiimide (DCC) were added to the anhydrous toluene solution containing compound A-E. Then the intermediate 3 was put into the reaction solution, and the reaction mixture was stirred at room temperature for 8–16 h to obtain **5a–5h**^34^.

**Table 1.** Structures of compounds **4a–4q**, **5a–5h**.

Product	R_1_	R_2_	R_3_	R_4_	R_5_	R_6_	R_7_	Yield (%)
**4a**	H	OH	H	H	F	H	H	70
**4b**	H	OH	H	H	CH_3_	H	H	58
**4c**	H	OH	H	H	CH_2_Cl	H	H	77
**4d**	H	OH	H	CH_3_	H	H	H	85
**4e**	H	OH	H	Cl	H	H	H	81
**4f**	H	OH	F	H	H	H	H	72
**4g**	H	OH	H	H	Cl	H	H	80
**4h**	H	OH	H	CH_3_	H	CH_3_	H	72
**4i**	OCH_3_	H	H	H	H	F	H	75
**4j**	OCH_3_	H	H	H	F	H	H	78
**4k**	OCH_3_	H	H	H	CF_3_	H	H	68
**4l**	OCH_3_	H	H	H	CH_2_Cl	H	H	70
**4m**	OCH_3_	H	H	CH_3_	H	H	H	87
**4n**	OCH_3_	H	F	H	H	H	H	82
**4o**	OCH_3_	H	OCH_3_	H	H	H	H	77
**4p**	OCH_3_	H	Cl	Cl	H	H	H	62
**4q**	OH	OH	H	H	CH_2_Cl	H	H	58
**5a**	H	OH						70
**5b**	OCH_3_	H						75
**5c**	H	OH						78
**5d**	OCH_3_	H						82
**5e**	H	OH						65
**5f**	OCH_3_	H						72
**5g**	OCH_3_	H						65
**5h**	OCH_3_	H						72

### Biology

#### Effect of compounds on proliferation of RA-FLSs

**Figure 1.**
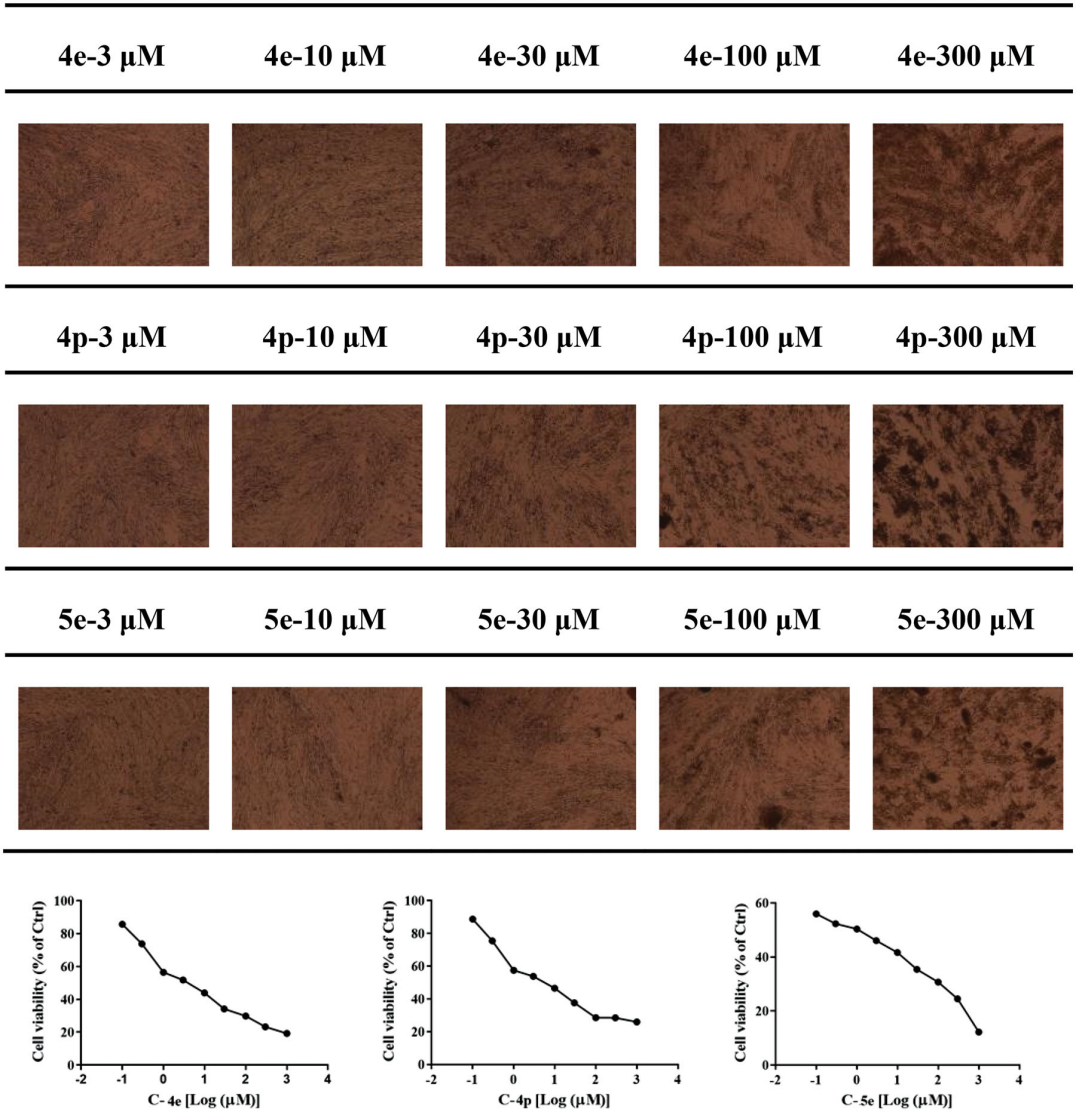
The cell proliferation inhibitory activity of compounds **4e**, **4p**, and **5e**.

Rheumatoid arthritis fibroblast-like synovial cells (RA-FLSs) are the main effector cells of RA, which exhibit abnormal activation and proliferation in synovial lesions. and mediate joint destruction and synovitis during RA pathological process^35^. Recent studies have found that RA-FLS cells activated in the chronic inflammatory environment have unique morphology and show characteristics similar to tumour cells, so the inhibition of RA-FLS cell proliferation is very important for the treatment of RA^36^. We first tested RA-FLS proliferation inhibitory activities of 25 3-(4-aminophenyl) coumarin derivatives using MTT assay with MTX as a reference compound. As shown in Table 2, some of the coumarin derivatives exhibited an inhibitory effect on RA-FLS proliferation. Among these compounds, heterocyclic substituted compound **5e**, with substitution of side-chain phenyl by naphthalene has the strongest activities (IC_50_ = 1.78 ± 0.02 µM), which was even stronger than MTX (IC_50_ = 5.00 ± 0.04 µM). When R_1_ is hydrogen and R_2_ is hydroxyl substitution, the activities of compound **4e** (IC_50_ = 5.60 ± 0.29 µM) and **4g** (IC_50_ = 9.13 ± 0.02 µM) are comparable to that of MTX. We, therefore, infer that R_4_ or R_5_ chlorination is significantly beneficial to the activity of RA-FLS proliferation inhibition. The activity of compound **4p** (IC_50_ = 7.61 ± 0.13 µM) is better than **4l** (IC_50_ = 15.71 ± 0.11 µM) and **4o** (IC_50_ = 16.57 ± 0.04 µM), suggesting that the RA-FLS inhibitory activity of R_2_ hydroxyl substituted compounds is better than that of R_1_ methoxy substituted compounds (Figure 1).

**Table 2.** Effect of compounds on proliferation of RA-FLSs. (mean ± SD, *n* = 3)

Compound	IC_50_ value (µM)	Compound	IC_50_ value (µM)
	RA-FLSs cell proliferation inhibitory activity		RA-FLSs cell proliferation inhibitory activity
**4a**	10.55 ± 0.28	**4n**	>50
**4b**	13.93 ± 0.02	**4o**	16.57 ± 0.04
**4c**	>50	**4p**	7.61 ± 0.13
**4d**	>50	**4q**	17.39 ± 0.33
**4e**	5.60 ± 0.29	**5a**	>50
**4f**	18.59 ± 0.04	**5b**	>50
**4g**	9.13 ± 0.02	**5c**	13.07 ± 0.02
**4h**	>50	**5d**	19.83 ± 0.03
**4i**	8.79 ± 0.19	**5e**	1.78 ± 0.02
**4j**	>50	**5f**	10.40 ± 0.32
**4k**	>50	**5g**	24.72 ± 0.03
**4l**	15.71 ± 0.11	**5h**	11.74 ± 0.29
**4m**	>50	**Methotrexate**	5.00 ± 0.04

#### Effect of compounds 4e, 4p, and 5e expression levels of RA-related cytokines

Cytokines are critical local protein mediators involved in almost all important biological processes, including cell growth, activation, and differentiation, inflammation, immunity^37^. It is well known that the imbalance between the activities of pro-inflammatory and anti-inflammatory cytokines in rheumatoid joints is conducive to the induction of autoimmunity and chronic inflammation, resulting in a joint injury. Therefore, the expression of related cytokines is closely related to the pathogenesis of RA^38^. Cytokines respond comprehensively to a variety of stimuli in the process of immunity and inflammation, involving both pro-inflammatory and anti-inflammatory activities. Cytokines such as IL-1 and tumour necrosis factor (TNF) have pro-inflammatory activity, while IL-10 mainly plays an anti-inflammatory role^39^. IL-1 is a prototype proinflammatory cytokine and has two forms, IL-1a and IL-1β, with indistinguishable biological activities in most studies^40^. IL-1 affects almost all cell types and usually acts synergistically with another pro-inflammatory cytokine, TNF. Although IL-1 is well known to up-regulate host defence and act as an immune adjuvant, it is also a highly inflammatory cytokine^41^. Inhibition of IL-1 was first applied for the treatment of RA. IL-1α is characterised by its immediate effect after release without further maturation. By binding to IL-1R1, IL-1α can trigger inflammation through induction of IL-1β activation and production of other cytokines, such as TNF or IL-6. IL-1β is the most functional member of the IL-1 family and high levels of IL-1β are found in synovium and fluid of RA patients^42^. As another important intercellular messenger, TNF-α has drawn more and more interest in the development of therapeutic methods for RA in recent years. It is generally believed that IL-1 plays a key role in joint destruction, while TNF-α is also involved in the systemic inflammatory process^43^. Numerous studies on synovial tissue of RA have demonstrated that TNF-α is a potential therapeutic target for RA, so the discovery of novel TNF-α inhibitors is of great significance for treatment of RA^44^^,^^45^.

**Figure 2.**
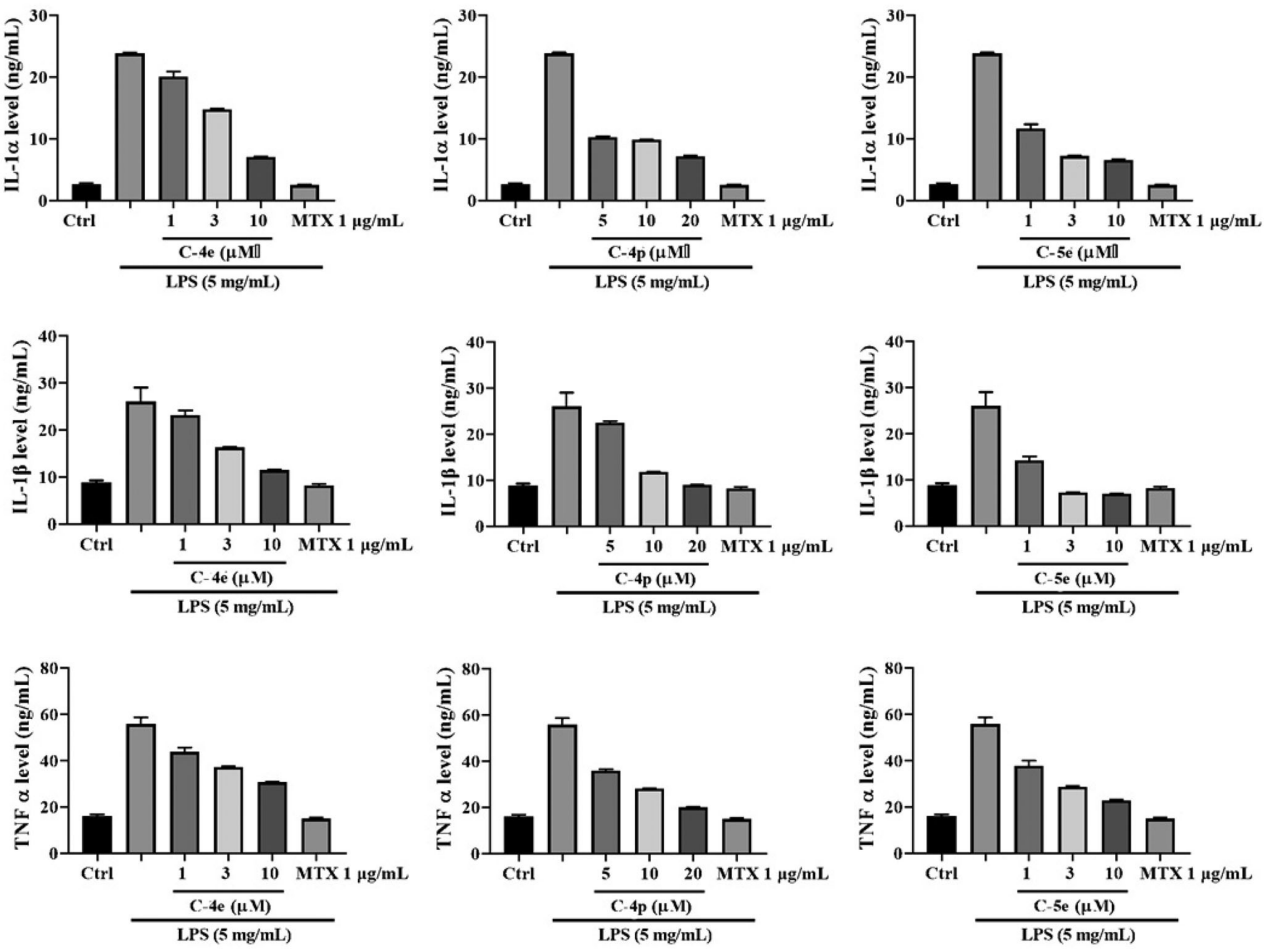
Effect of compounds **4e**, **4p**, and **5e** on secretion of IL-1α, IL-1β, and TNF-α.

Compounds **4e** (IC_50_ = 5.60 ± 0.29 µM), **4p** (IC_50_ = 7.61 ± 0.13 µM), and **5e** (IC_50_ = 1.78 ± 0.02 µM) showed an excellent inhibitory effect on RA-FLS proliferation. We next used these optimised compounds to further explore their anti-RA activities and related mechanisms. We first detected the effect of these compounds on the contents of cytokine IL-1α, IL-1β, and TNF-α released by RA-FLSs with stimulation LPS. The results showed that the three compounds could concentration-dependently reduce the secretion of IL-1α, IL-1β, and TNF-α. Compound **5e** exhibited stronger activity than Compounds **4e** and **4p** (Figure 2).

RA-FLS cells were cultured at 37 °C for 24 h, then the cells were divided into four groups: blank control group, model group (LPS), LPS + compound group, and LPS + MTX group. Different concentrations of compounds and 1.0 µg/mL MTX, were added to incubate for 48 h, and then LPS was added to stimulate the secretion of proinflammatory cytokines at the final concentration of 5 µg/mL. After 24 h of incubation, pro-inflammatory cytokines including IL-1 α, IL-1 β, and TNF-α were measured by ELISA method.

**Figure 3.**
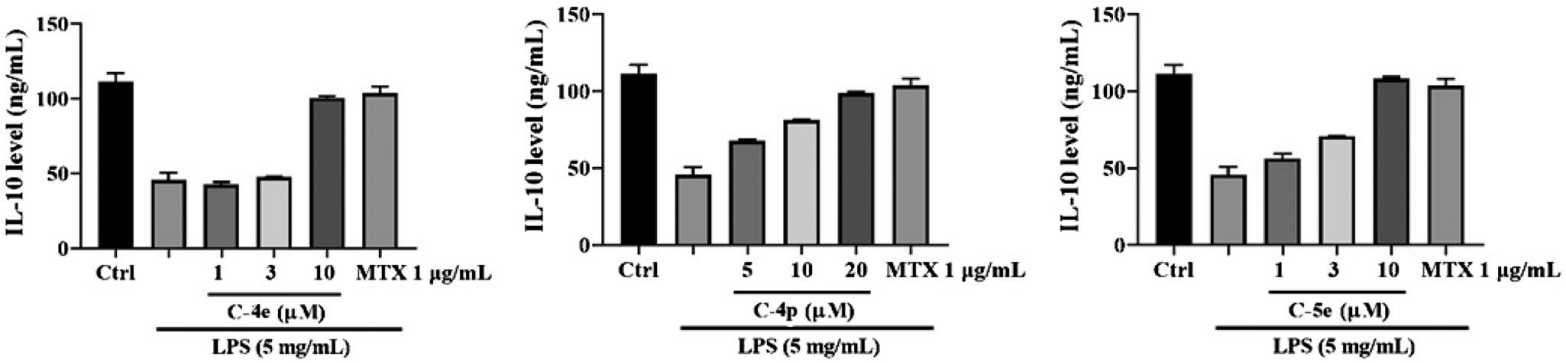
Effect of compounds **4e**, **4p**, and **5e** on secretion of cytokine IL-10.

Multiple cytokine IL-10 is an effective inhibitor in immune cells including macrophages, T cells and NK cells, and it can block the synthesis and activation of cytokines in these cells. Different from pro-inflammatory factors IL-1 and TNF-α, IL-I0 has effective anti-inflammatory properties^46^^,^^47^. We examined the effects of **4e**, **4p**, and **5e** on cytokine IL-10. The results showed that all the derivatives could increase the amount of IL-10, which preliminarily indicated that the derivatives were beneficial to the fight against RA disease (Figure 3).

We also detected the effect of these compounds on the content of IL-10 released by RA-FLSs stimulated by LPS, and the results showed that **4e**, **4p**, and **5e** could significantly increase the content of IL-10 released by RA-FLSs. These results indicate that compounds **4e**, **4p**, and **5e** could inhibit the production of proinflammatory cytokines and enhance the production of anti-inflammatory cytokines, therefore exert anti-inflammatory functions. Compound **5e** has the strongest activity among them, which might be a potential anti-RA drug candiate^48^.

#### Effect of compound 5e on luciferase activity of NF-κB

**Figure 4.**
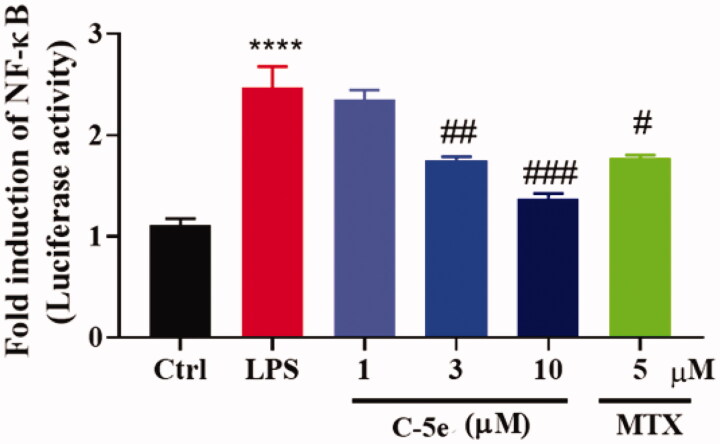
Inhibitory effect of compound **5e** on luciferase activity of NF-κB. RA-FLS cells were cultured at 37 °C for 24 h, then NF-κB luciferase plasmid and control sea kidney plasmid were transfected for 48 h. Different concentrations of compound **5e** and LPS (final concentration: 5 μg/mL) were added to stimulate the secretion of proinflammatory cytokines. After incubation for 48 h, the cells were collected and the luciferase activity of NF-κB pathway was detected by dual luciferase system. ^#^*p* < 0.05, ^##^*p* < 0.01, ^###^*p* < 0.001, compared with model group; ****p < 0.0001 compared with ctrl group.

The activation of a series of signal pathways is a typical feature of chronic synovitis in RA. These signal pathways induce phosphorylation of cytoplasmic proteins in synovial cells, and phosphorylation of transcription factors and nuclear proteins such as NF-κB^49^, thus promoting cell proliferation and activation. On the other hand, inflammatory factors such as TNF-α, IL-1α, and IL-1β in turn regulate cell membrane receptors and activate the mitogen-activated protein kinase (MAPK) signal pathway. Therefore, investigation of the above signalling events is of great significance for the in-depth understanding of the pathogenesis of RA and the development of new drugs for treatment. NF-κB-Luc luciferase reporter gene plasmid is a reporter gene used to detect the transcriptional activity of NF-κB. The enhanced activity of NF-κB stimulated by LPS leads to a significant increase in the expression of inflammatory proteins. We next treated RA-FLSs with compound **5e** and tested the luciferase activity of NF-κB. Similar to MTX, compound **5e** can significantly reduce the activity of NF-κB, which is of great significance in inhibiting inflammatory factors and reducing inflammation (Figure 4).

#### Effect of compound 5e on MAPK signal pathway

**Figure 5.**
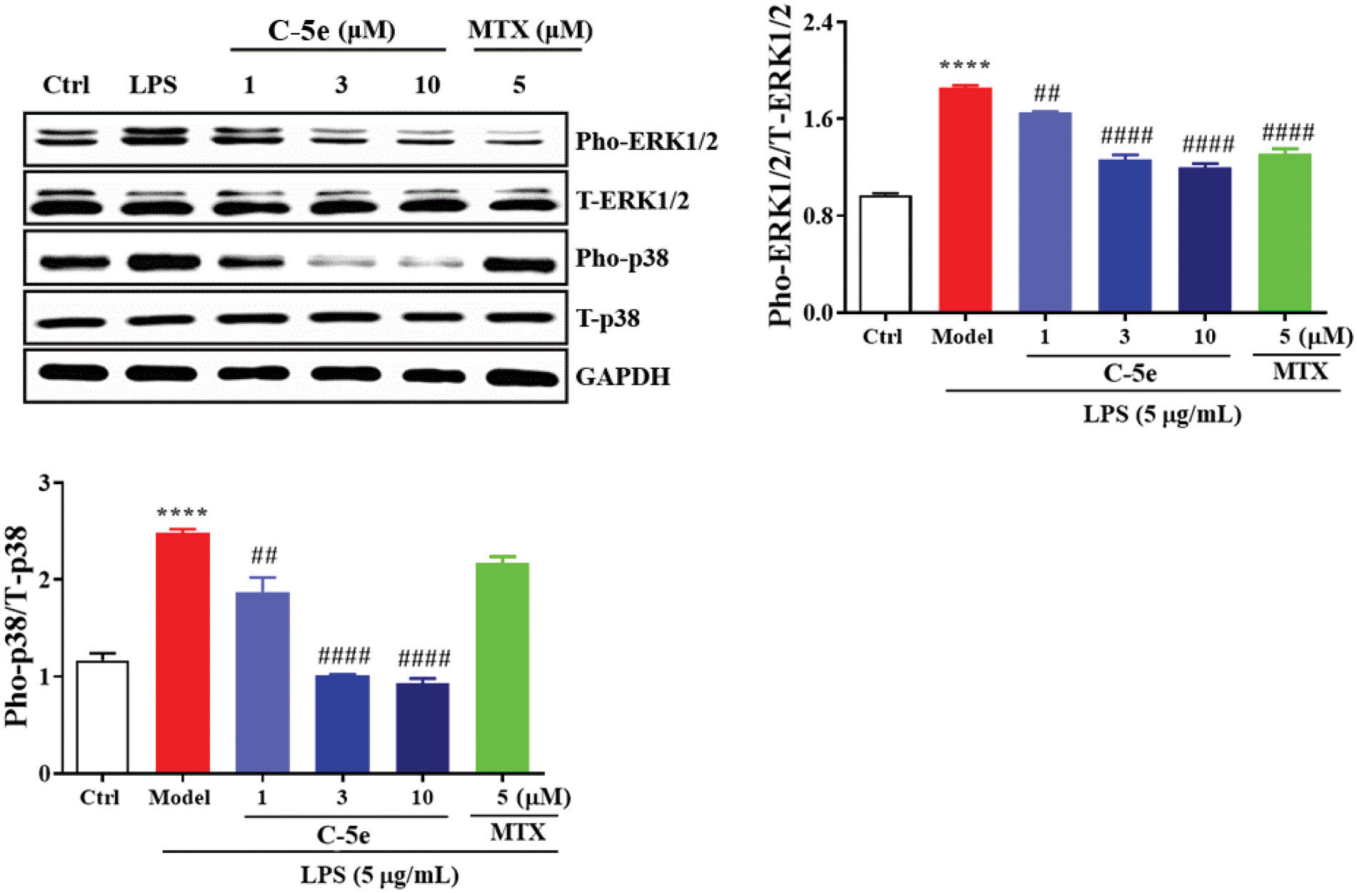
Inhibitory effect of compound **5e** on MAPK signal pathway. RA-FLS cells were cultured at 37 °C for 24 h. The cells were preincubated with compound **5e** of different concentrations for 1 h, and then treated with LPS (final concentration: 5 μg/mL) for 72 h. Collected cells were used for Western blotting analysis of anti-ERK1/2, anti-pho-ERK1/2, anti-p38 and anti-pho-p38 antibodies. ^##^*p* < 0.01, ^####^*p* < 0.0001 compared with model group; ****p < 0.0001 compared with ctrl group.

MAPK mediates important pathways in the eukaryotic signal transduction network, which plays a key role in regulating gene expression and promoting disease development^50^^,^^51^. In mammals, five different MAPK-mediated signal pathways regulate cell growth and differentiation. JNK and p38 MAPK signalling pathways play an important role in stress responses such as inflammation and apoptosis^52^. We next treated RA-FLSs with compound **5e** and tested the changes in MAPK-mediated signal pathways. The results showed that LPS stimulation significantly increased the levels of pho-extracellular signal-regulated kinases (ERK)1/2 and pho-p38, and compound **5e** could significantly reduce the levels of pho-ERK1/2 and pho-p38. These results indicate that compound **5e** can effectively suppress the phosphorylation and activation of ERK1/2 and P38 in the MAPK signal pathway (Figure 5), which is well known to cause suppression of NF-κB activity. In contrast, MTX only suppressed the phosphorylation of ERK1/2 but not the p38.

#### Anti-RA activity of compound 5e in vivo

**Figure 6.**
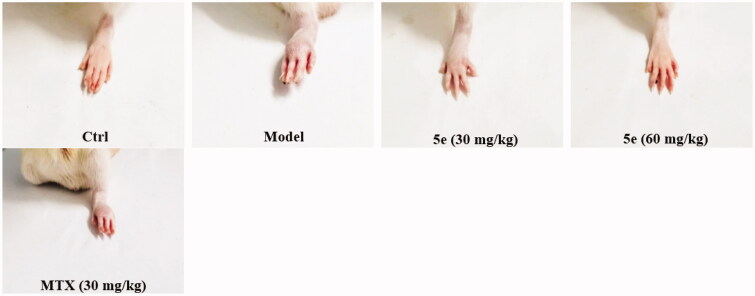
Establishment and treatment of CFA rat model of RA. Normal control group (Ctrl, saline), model group (Model, saline), high dose compound **5e** (60 mg/kg) group, low dose compound **5e** (30 mg/kg) group, positive control MTX (30 mg/kg) group. (*n* = 5 per group).

**Figure 7.**
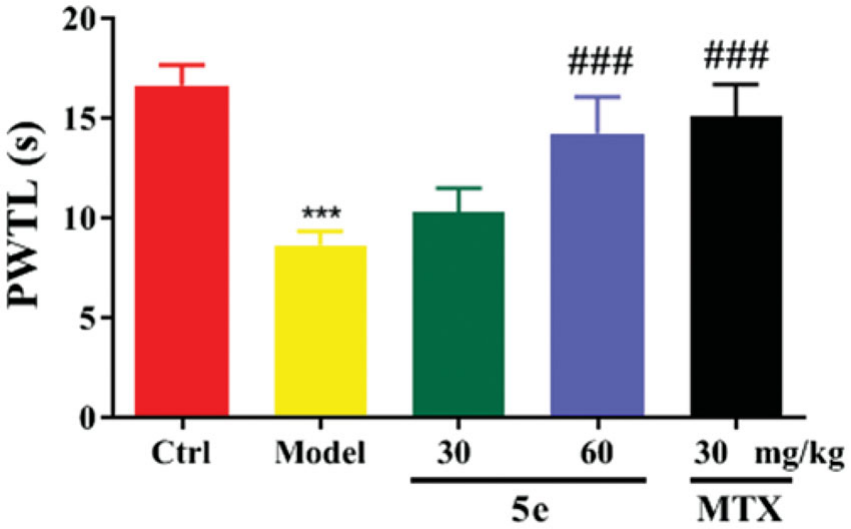
The analgesic effect of compound **5e** on rats was detected by the method of thermal pain threshold of rat claws. ^###^*p* < 0.001, ***p < 0.001. (*n* = 5 per group).

**Figure 8.**
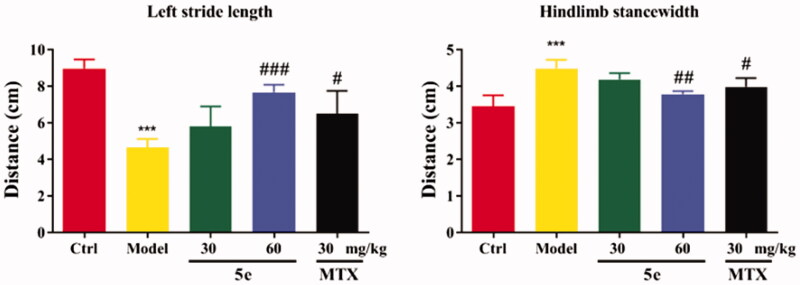
Motor function test of SD rats. Place the rat in the plexiglass chamber of the treadmill (25 cm long and 5 cm wide), set the treadmill speed to 18 cm/s, the shutter speed to take pictures: 40 ms, and record 5 s for each animal. The motor function of RA rats was explained by the motor parameters of left stride length and hindlimb stance width. ^#^*p* < 0.05, ^##^*p* < 0.01, ^###^*p* < 0.001, compared with model group, ***p < 0.001 compared with ctrl group. (*n* = 5 per group).

To establish an arthritis model, we used Freund’s complete adjuvant to induce arthritis in rats via injection of CFA into the left hind paw of rats^53^^,^^54^. After injection of CFA into SD rats, the results showed that 80–90% of the rats showed paw swelling within 14–28 days (Figure 6, model group). The model rats were treated with compound 5e of different concentrations once a day from day 14 to day 28. MTX was used as a positive control. The experimental results are shown in Figure 6. After successfully establishing the model, we tested the pain threshold and motor function of RA rats. The reflex latency (paw withdraw thermal latency, PWTL) of rat foot contraction induced by thermal stimulation was used as an index of hindlimb pain threshold by the hot plate method. As shown in Figure 7, the PWTL of the model group was shorter than that of the control group (*p* < 0.001). After the intervention of compound **5e** in the low-dose group and high-dose group, the results showed that the PWTL of RA rats was prolonged, and the prolongation of PWTL was positively correlated with the drug concentration^55^. It is proved that compound **5e** reduces thermal hyperalgesia in RA rats and has an analgesic effect on RA rats. In the motor function test, the left gait width and hind limb gait width of RA rats before and after treatment were measured by gait analysis. Compared with the control group, the left stride length of the model group was shorter, and the hindlimb stance width was wider, and the motor function of rats was blocked (*p* < 0.001). After treatment with compound **5e** in low-dose group and high-dose group, it was found that compound **5e** could improve the exercise ability of rats (Figure 8).

**Figure 9.**
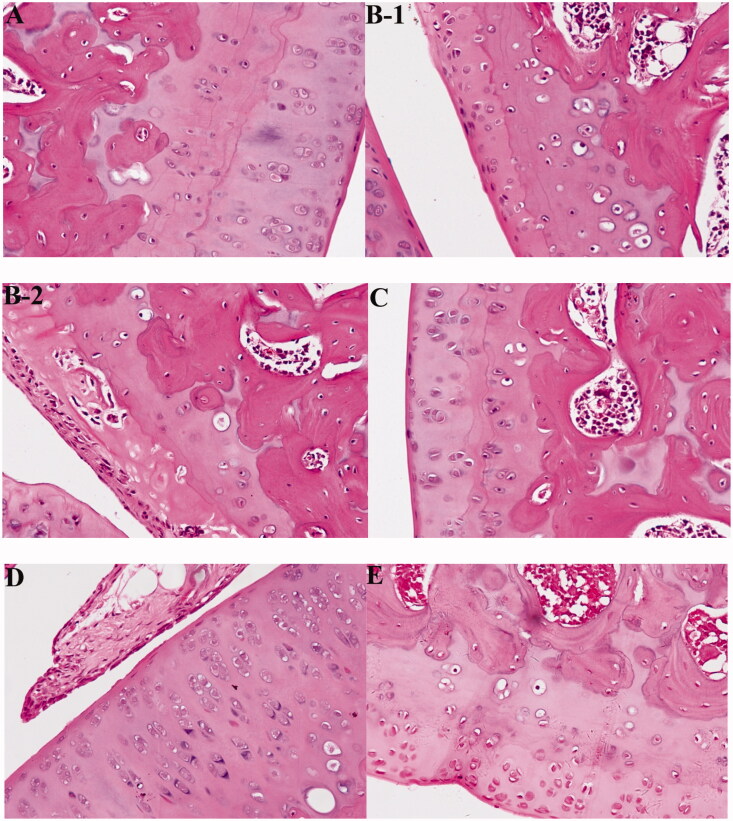
Compound **5e** recovers RA histopathological features of rats. (A) Normal control group (Ctrl, normal saline), (B-1, B-2) model group (model, normal saline), (C) low-dose compound **5e** group (30 mg/kg), (D) high-dose compound **5e** Group (60 mg/kg), and (E) positive control MTX (30 mg/kg). (*n* = 5 per group).

We then treated RA rats with compound **5e**. In normal rats, no inflammation pathological features were observed, the surface of cartilage was smooth and free of cracks, and the cells were evenly distributed (Figure 9(A)). In the arthritis model group, obvious inflammation pathological features were observed, including disorder of chondrocytes and vacuolisation of some cells, which indicate decreased activity or died cells, and destroyed cartilage surface (Figure 9(B-1,B-2)). After treatment with 30 mg/kg of compound **5e**, the inflammation was significantly improved, the vacuole of chondrocytes decreased, and the destruction of the cartilage surface was recovered (Figure 9(C)). After treatment with 60 mg/kg of compound **5e**, almost no inflammatory cell infiltration was observed, the surface of cartilage tended to be smooth, indicating a better therapeutic effect than low dose compound **5e** (Figure 9(D)).

## Conclusion

In this study, we screened the anti-RA activity of a series of 3-(4-aminophenyl)-coumarins synthesised by our team. Compound **5e** had the strongest inhibitory effect on the proliferation of RA-FLS_S_. We further discussed the anti-inflammatory activity of compound **5e** and its related mechanism. We found that compound **5e** could inhibit the expression of inflammation-related cytokines (IL-1 α, IL-1 β, and TNF-α) induced by lipopolysaccharide. The preliminary mechanism study found that compound **5e** has an effect on inflammation-related signal transduction, which can inhibit the activity of nuclear factor-mitogen-activated protein B (NF-κB) through mitogen-activated protein kinase (MAPK) signal pathway. Finally, the rat arthritis model established by Freund’s complete adjuvant further proved that compound **5e** had anti-RA activity *in vivo*. To sum up, compound **5e** may be a potential protective compound for the treatment of RA.

## Experimental

### Materials and methods

#### Materials

Methotrexate, manufacturer: MCE. Bio Legend LEGEND plex TM multiplex bead-based assay (# 740007), brand: Biolegend; manufacturer: Biolegend Corporation of the United States. Saline, manufacturer: Guangdong Litai Pharmaceutical Co., Ltd. PBS powder, manufacturer: American SIGMA Company.

#### Cells and cell culture

RA-FLS cells were obtained from Beijing Luyuan Byrd Biotechnology Co., Ltd., and the 3rd–5th generations were used in the experiments. The cells were cultured with high glucose Dulbecco’s Modified Eagle Medium (DMEM) supplemented with 10% foetal bovine serum (FBS), 100 units/mL penicillin, 100 µg/mL streptomycin, and 2 mM glutamine. All cells were incubated in a humidified atmosphere of 95% air and 5% CO_2_ at 37 °C.

#### MTT assay

RA-FLS cells were inoculated in a 96-well plate (5 × 10^−3^/well/100 µL), incubated for 24 h, and then treated with different concentrations of synthesised derivatives (0.1, 0.3, 1, 3, 10, 30, 100, and 300 µM) or 1.0 µg/mL MTX for 72 h. The culture medium, was then removed and the cells were incubated with 5 µg/mL MTT at 37 °C for 4 h, and 100 µL DMSO was added to each well to dissolve the formed methylazan The absorbance (OD value) was measured at 570 nm using a plate reader. The cell proliferation inhibition rate was calculated by following the formula: inhibition rate = (OD value of the control group-OD value of the treated groups)/OD value of the control group × 100%. The IC_50_ value (µM) was calculated according to the curve.

#### Test of cytokines using enzyme-linked immunosorbent assay (ELISA)

The 3rd–5th passage RA-FLS cells were inoculated in 48-well plates (1.5 × 10^6^/well) and cultured at 37 °C for 24 h, and then cells were divided into four groups: blank control group, model group (LPS), LPS + compound group and LPS + MTX group. Different concentrations of compounds and 1.0 µg/mL MTX were added and incubated for 48 h, and then LPS was added at a final concentration of 5 µg/mL to stimulate the secretion of proinflammatory cytokines. After a 24 h incubation, the cell culture medium of each group was collected, and the cell fragments were removed by centrifugation at 10,000 × *g* for 5 min at 4 °C. The proinflammatory cytokines including IL-1α, IL-1β, TNF-α, and IL-10 were tested using ELISA assay by following the manufacturer’s instructions.

#### Test of luciferase activity of NF-κB

RA-FLS cells were seeded in 24-well plate (1 × 10^5^/well) and cultured at 37 °C for 24 h, then NF-κB luciferase plasmid and control renilla plasmid were transfected for 48 h. Different concentrations of compound **5e** and LPS (final concentration: 5 µg/mL) were added to stimulate the secretion of proinflammatory cytokines. After a 48-h incubation later, the cells were collected and the luciferase activity of NF-κB pathway was detected using the double luciferase system.

#### Test of MAPK signal pathway

RA-FLS cells (3 × 10^5^/well/2 mL) were seed in 6-well plate cultured at 37 °C and for 24 h. The cells were pre-incubated with different concentrations of compound **5e** for 1 h, and then were treated with LPS (final concentration: 5 µg/mL) for 72 h. The cells were collected for Western blotting analysis using anti-ERK1/2, anti-pho-ERK1/2, anti-p38, and anti-pho-p38 antibodies.

#### Western blotting

Cells were lysed in 20 mM HEPES, pH 7.5, 150 mM NaCl, 1% NP-40, 10 mM tetrasodium pyrophosphate, 100 mM NaF, 17.5 mM 1-glycerophosphate buffer supplemented with Complete Mini Protease Inhibitor Cocktail tablets (Roche Applied Sciences, Indianapolis, IN). Samples separated by SDS-PAGE were transferred to nitrocellulose membranes, blocked with 5% bovine serum albumin (w/v) at room temperature for 1 h, and incubated with primary antibodies (1:1000 dilution) at 4 °C overnight. After incubation with secondary antibody (1:3000 dilution) at room temperature for 1 h, the membranes were developed with chemiluminescence ECL reagent (Lumi Gold, Signa Gen Laboratories, Rockville, MD) and exposed to Hyper film MP (GE Healthcare Life Science, Pittsburgh, PA).

#### Animals

SD rats weighing 180–220 g were obtained from Jinan Peng Yue Experimental Animal Co., Ltd. (license No.: SCXK (Lu) 2014 Mel 0007), Co., Ltd. The animals were kept in captivity under standard laboratory conditions and ingested standard pellet feed and water at will. All experiments involving live animals and their care are conducted in strict accordance with the “National Care and use of Experimental Animals” of the State Animal Research Bureau (China) and the “regulations on the Administration of Experimental Animals” of the Life Science Research Centre of Shandong first Medical University. The experiment was approved by the Animal Protection and use Committee of Shandong first Medical University. Make every effort to reduce the suffering of animals and reduce the number of animals used.

#### Establishment and treatment of adjuvant arthritis in rats

The rat adjuvant arthritis model was first established by Freund in the 1950s. Which is simple and selective and is a widely used immune inflammation model^56^^,^^57^. 8-week-old Sprague–Dawley (SD) rats, weighing 180–220 g, were purchased from Jinan Peng Yue Experimental Animal Breeding Co., Ltd., Jinan, China. The Freund’s complete adjuvant (CFA) was purchased from Sigma-Aldrich (item number and specification: F5881-10 mL) and 0.1 mL CFA was injected into the left hind paw of rats to induce inflammation. Arthritis was evaluated by observing the changes in paw swelling every week. After the rat adjuvant arthritis model was successfully established, SD rats were divided into the following groups: normal control group (Ctrl, saline), model group (Model, saline), high dose compound **5e** (60 mg/kg) group, low dose compound **5e** (30 mg/kg) group, positive control MTX (30 mg/kg) group, (*n* = 5 per group). The compounds solutions (1 mL) or saline were orally administered to rats once a day for four weeks. Then the mice were euthanized using carbon dioxide (CO_2_) and the knee joints were harvested and analysed.

#### Heat pain threshold determination

The thermal pain threshold of rats was measured by rat claw thermal radiation stimulation pain metre (7371 plantar test, Ugo Basile, Italy). The rats are in a state of free activity, adjust the instrument stimulation intensity (IR = 72), put the left hindlimb on the instrument, and measure the paw recovery time of rats. The reflex latency (paw withdrawal thermal latency, PWTL) of foot contraction induced by thermal stimulation at the bottom of rat foot is the thermal pain threshold.

#### Rat motor function test

The automatic gait analysis system (DigiGait^®^, Mouse Specific, Inc.)) was used to test the rats. The rats were placed in the plexiglass chamber of the treadmill (25 cm long and 5 cm wide). The plastic rear bumper of the rats was used to tap the rats to keep them in the video shooting range. The treadmill speed was set to 18 cm/s. The shutter speed was 40 ms, and each animal recorded 5 s. DigiGait software automatically recognises paw prints and analyses gait parameters such as step size and step width.

#### Immunohistochemistry (IH)

The rat knee joints were fixed in phosphate-buffered formalin, embed in paraffin, cut in 4 µm thickness, and applied to slides. The slides were deparaffinized in xylenes using three changes for 5 min each and hydrated gradually through graded alcohols: 100% ethanol twice for 10 min each, 95% ethanol twice for 10 min each, and then deionised water for 1 min with stirring. For antigen unmasking, slides were placed in a container, covered with 10 mM sodium citrate buffer, pH 6.0, and heated in a convection steamer for 1 h. The slides were washed in deionised water three times for 2 min each, blocked with 5% normal goat blocking serum for 30 min, and then stained with haematoxylin and eosin (HE). The slides were analysed and photographed using a micromirror.

#### Statistics

*t*-Test and analysis of variance (ANOVA) were performed using StatView (SAS Institute, Cary, NC). The data shown are representatives of at least two independent experiments with similar results, and the data points represent the mean of at least triplicate measurements with error bars corresponding to standard deviation. *p* < 0.05 was considered significantly different.
